# Supplementary material for: Mechanistic Chromatographic Column Characterization for the Analysis of Flavonoids Using Quantitative Structure-Retention Relationships Based on Density Functional Theory
Source: Int J Mol Sci. 2020 Mar 17;21(6):2053. doi: 10.3390/ijms21062053 (PMC7139519; doi:10.3390/ijms21062053)
Supplement: Supplementary file 1 [file ijms-21-02053-s001.zip › supplementary/supplementary_QSRR_IJMS.docx]

Article

Mechanistic chromatographic column characterization for the analysis of flavonoids using quantitative structure-retention relationships based on density functional theory

Bogusław Buszewski ^1, 2,^ *, Petar Žuvela ^3^, Gulyaim Sagandykova ^1, 2^, Justyna Walczak-Skierska ^2^, Paweł Pomastowski ^2^, Jonathan David ^3^, Ming Wah Wong ^3,^ *

^1^ Department of Environmental Chemistry and Bioanalytics, Faculty of Chemistry, Gagarina 7, 87-100, Toruń, Poland.; sagandykova.gulyaim1@gmail.com (G.S.)

^2^ Interdisciplinary Centre for Modern Technologies, Nicolaus Copernicus University, Wileńska 4, 87-100, Toruń, Poland; pawel_pomastowski@wp.pl (P.P.), walczak-justyna@wp.pl (J.W.)

^3^ Department of Chemistry, National University of Singapore, 3 Science Drive 3, 117543, Singapore, petar.zuvela@nus.edu.sg; jonathan.david14@sps.nus.edu.sg (J.D.)

***** Correspondence: bbusz@chem.umk.pl and chmwmw@nus.edu.sg

Received: date; Accepted: date; Published: date

**Contents:**

**Ref S1.** Full Gaussian 16 citation.

**Figure S1.** MRM transitions and retention times for the flavonoids analyzed on the conventional Kinetex C-18 column.

**Figure S2.** MRM transitions and retention times for the flavonoids analyzed on the Kinetex F5 column.

**Figure S3.** MRM transitions and retention times for the flavonoids analyzed on the IAM.PC.DD2 column.

**Figure S4.** Optimization of latent variables for the final consensus GA-PLS models for: A) Kinetex C-18 column, B) Kinetex F5 column, C) IAM.PC.DD2 column.

**Figure S5.** Graphical depiction of the optimized 3D molecular structures of all the flavonoids.

**Table S1.** Summary of the experimental retention values of flavonoids on three stationary phases and the values of the QM parameters employed for QSRR modelling. (uploaded as a separate supporting spreadsheet file).

**Ref S1.** Gaussian 16, Revision B.01, Frisch, M. J.; Trucks, G. W.; Schlegel, H. B.; Scuseria, G. E.; Robb, M. A.; Cheeseman, J. R.; Scalmani, G.; Barone, V.; Petersson, G. A.; Nakatsuji, H.; Li, X.; Caricato, M.; Marenich, A. V.; Bloino, J.; Janesko, B. G.; Gomperts, R.; Mennucci, B.; Hratchian, H. P.; Ortiz, J. V.; Izmaylov, A. F.; Sonnenberg, J. L.; Williams-Young, D.; Ding, F.; Lipparini, F.; Egidi, F.; Goings, J.; Peng, B.; Petrone, A.; Henderson, T.; Ranasinghe, D.; Zakrzewski, V. G.; Gao, J.; Rega, N.; Zheng, G.; Liang, W.; Hada, M.; Ehara, M.; Toyota, K.; Fukuda, R.; Hasegawa, J.; Ishida, M.; Nakajima, T.; Honda, Y.; Kitao, O.; Nakai, H.; Vreven, T.; Throssell, K.; Montgomery, J. A., Jr.; Peralta, J. E.; Ogliaro, F.; Bearpark, M. J.; Heyd, J. J.; Brothers, E. N.; Kudin, K. N.; Staroverov, V. N.; Keith, T. A.; Kobayashi, R.; Normand, J.; Raghavachari, K.; Rendell, A. P.; Burant, J. C.; Iyengar, S. S.; Tomasi, J.; Cossi, M.; Millam, J. M.; Klene, M.; Adamo, C.; Cammi, R.; Ochterski, J. W.; Martin, R. L.; Morokuma, K.; Farkas, O.; Foresman, J. B.; Fox, D. J. Gaussian, Inc., Wallingford CT, 2016.

**Figure S1**. MRM transitions and retention times for the flavonoids analyzed on the conventional Kinetex C-18 column.

**Figure S2**. MRM transitions and retention times for the flavonoids analyzed on the Kinetex F5 column.

**Figure S3**. MRM transitions and retention times for the flavonoids analyzed on the IAM.PC.DD2 column.


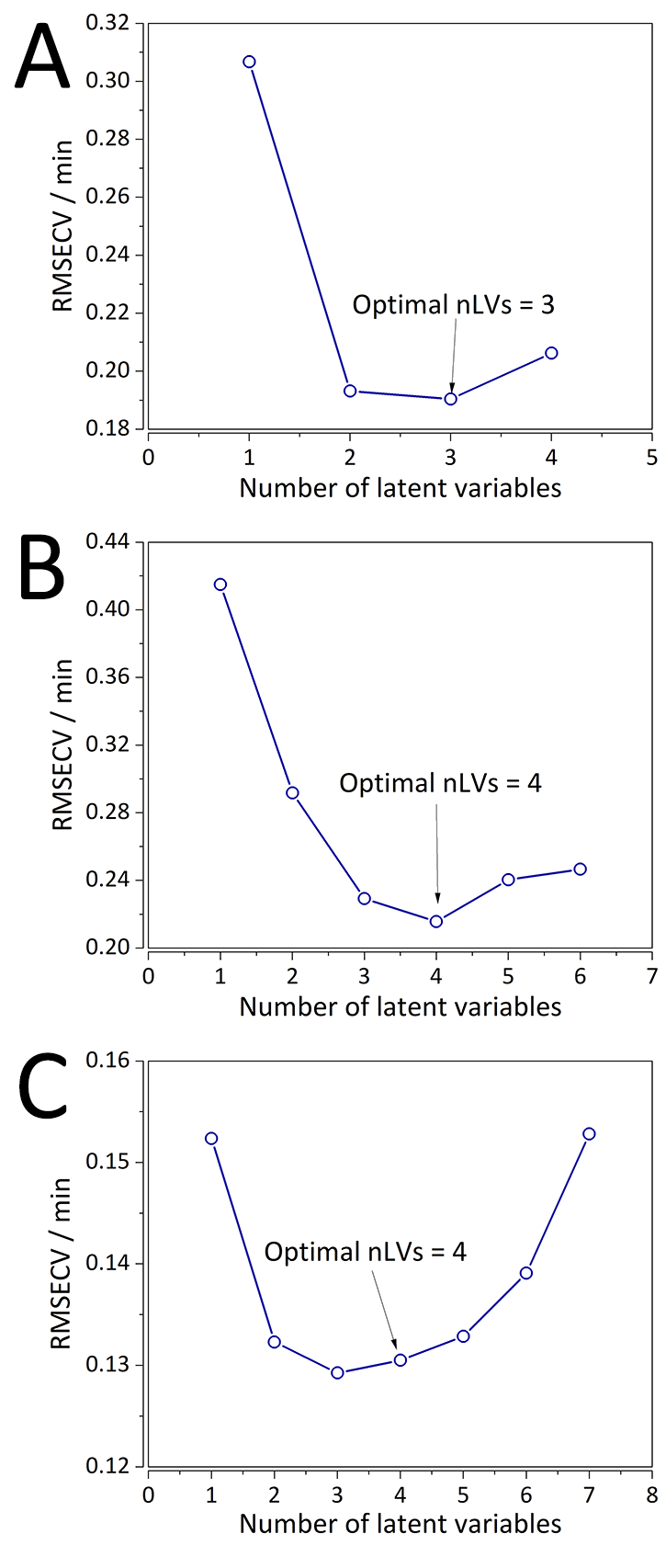


**Figure S4.** Optimization of latent variables for the final consensus GA-PLS models for: **A)** Kinetex C-18 column, **B)** Kinetex F5 column, **C)** IAM.PC.DD2 column.


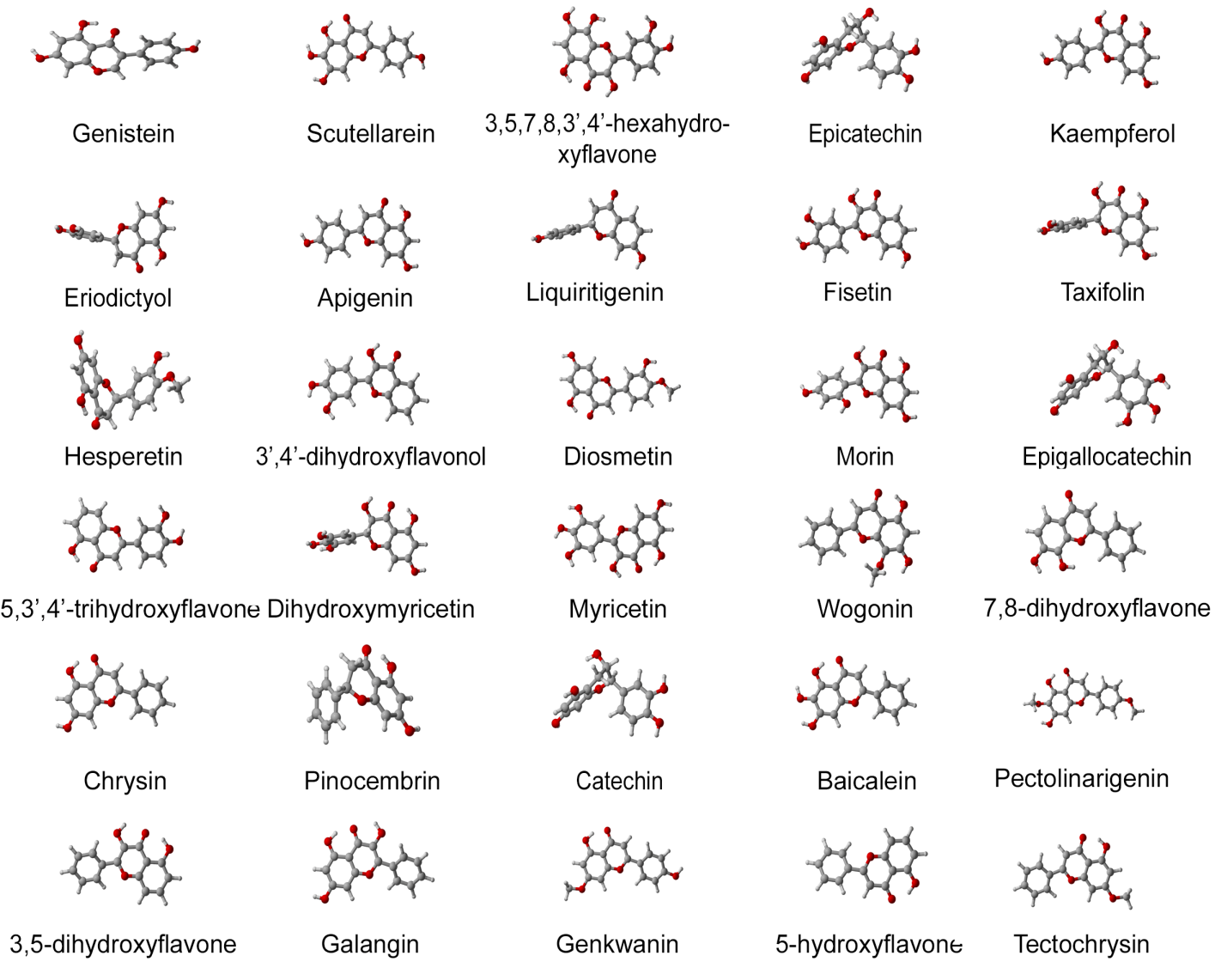


**Figure S5.** Graphical depiction of the optimized 3D molecular structures of all the flavonoids.
